# Supplementary material for: Deletion of skeletal muscle Akt1/2 causes osteosarcopenia and reduces lifespan in mice
Source: Nat Commun. 2022 Oct 5;13:5655. doi: 10.1038/s41467-022-33008-2 (PMC9535008; doi:10.1038/s41467-022-33008-2)
Supplement: Supplementary file 1 — Supplementary Information [file 41467_2022_33008_MOESM1_ESM.pdf]

Supplementary Information for

Deletion of skeletal muscle *Akt1/2* causes osteosarcopenia and reduces lifespan in mice

Sasako T, et al.

This PDF file includes Supplementary Figures 1 to 11

Supplementary Figure 1

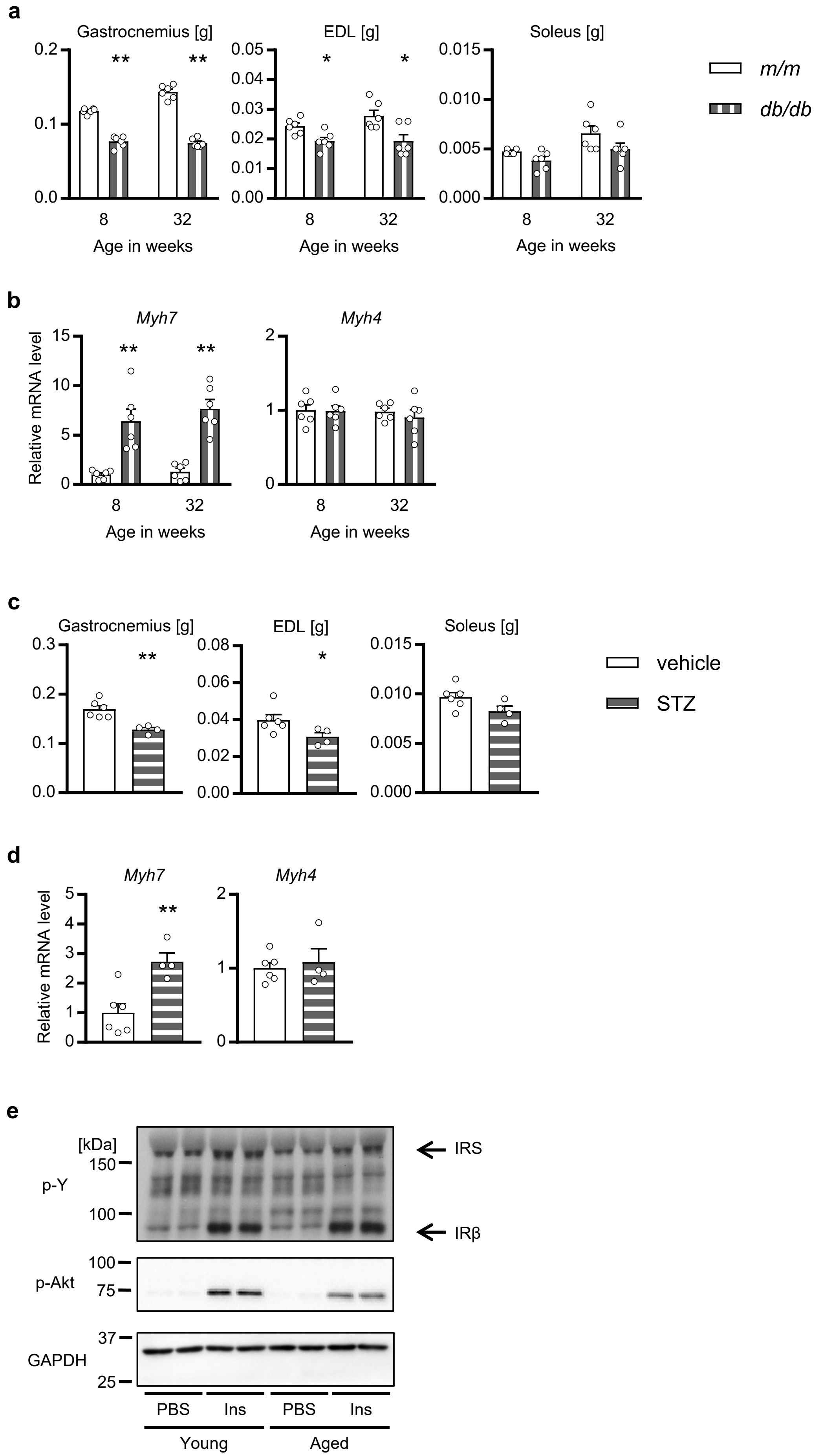

Supplementary Fig. 1 Effects of diabetes and aging on skeletal muscle.

a-d, (**a,c**) Skeletal muscle weight, and (**b,d**) gene expression of myofiber markers in EDL analyzed by RT-PCR of, (**a,b**) *db/db* mice at the age of the indicated weeks (n = 6 mice per group, as shown by the number of data points on the graph, same as below), and (**c,d**) wild type mice 4 weeks after treatment with streptozotocin (STZ) (n = 4 or 6 mice).

e, Representative images of western blotting to analyze insulin signaling in gastrocnemius of C57BL/6J mice at the age of 8 weeks (young) and 55 weeks (old) after treatment with insulin for 10 minutes. The experiments were repeated independently twice.

Values of the data are expressed as mean ± SEM. IRβ: insulin receptor β subunit, IRS: insulin receptor substrates, *$P < 0.05$, **$P < 0.01$. Unpaired 2-tailed t-test was used for assessment, and the exact P values are provided in Supplementary Data 3. Source data are provided as a Source Data file.

Supplementary Figure 2

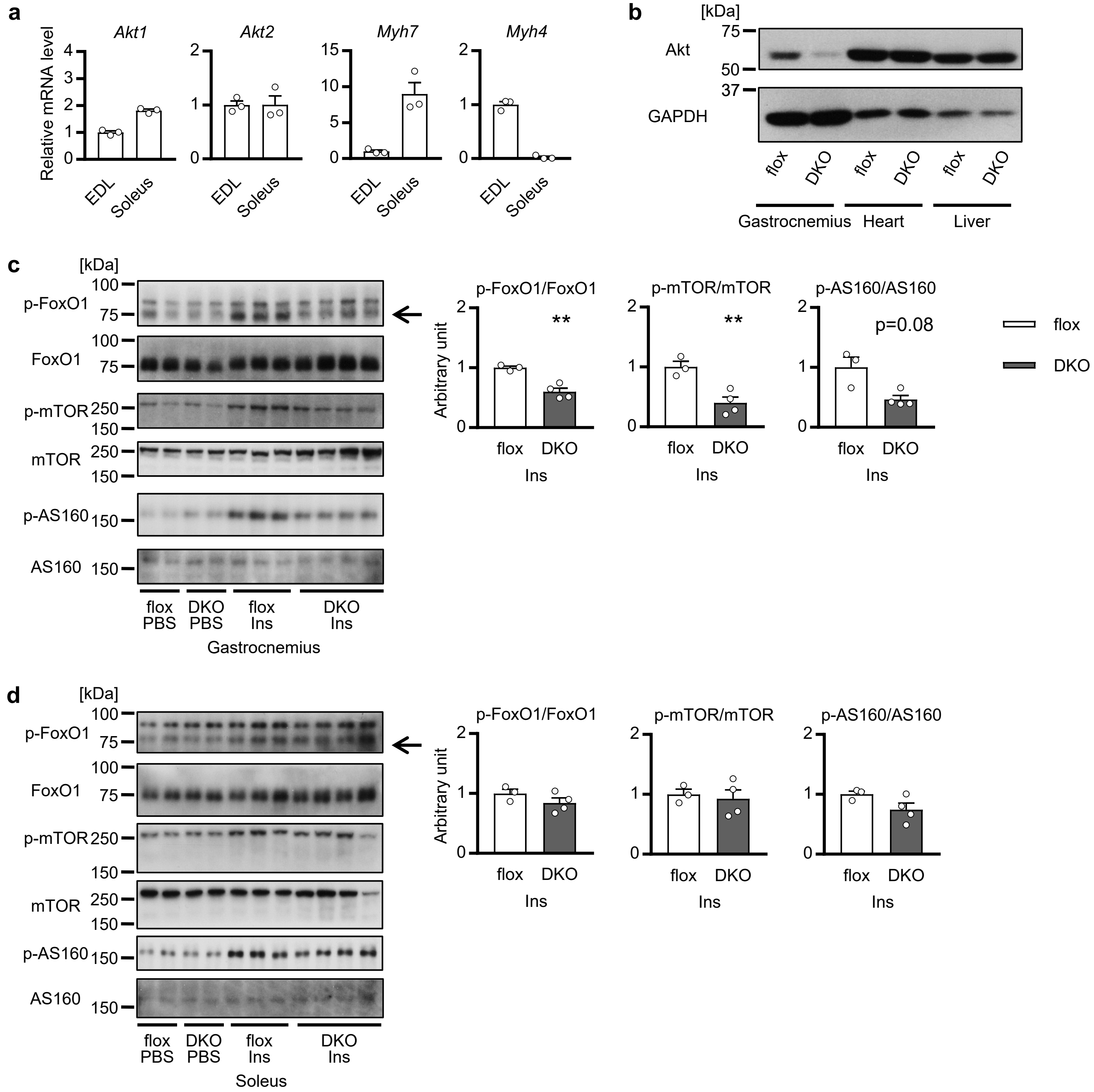

Supplementary Fig. 2 Akt expression in the mAktDKO mice.

a, Gene expression of Akt isoforms and myofiber markers in EDL and soleus of wild type mice at the age of 8 weeks analyzed by RT-PCR (n = 3 mice per group, as shown by the number of data points on the graph, same as below).

b, Representative images of western blotting to analyze expression of Akt protein in tissues of the mAktDKO mice at the age of 8 weeks. The experiments were repeated independently twice.

c,d, Insulin signaling in, (**c**) gastrocnemius, and (**d**) soleus, of the mAktDKO mice at the age of 8 weeks after treatment with insulin for 10 minutes analyzed by western blotting (n = 3 or 4 mice).

Values of the data are expressed as mean ± SEM. **$P < 0.01$. Unpaired 2-tailed t-test was used for assessment, and the exact P values are provided in Supplementary Data 3. Source data are provided as a Source Data file.

Supplementary Figure 3

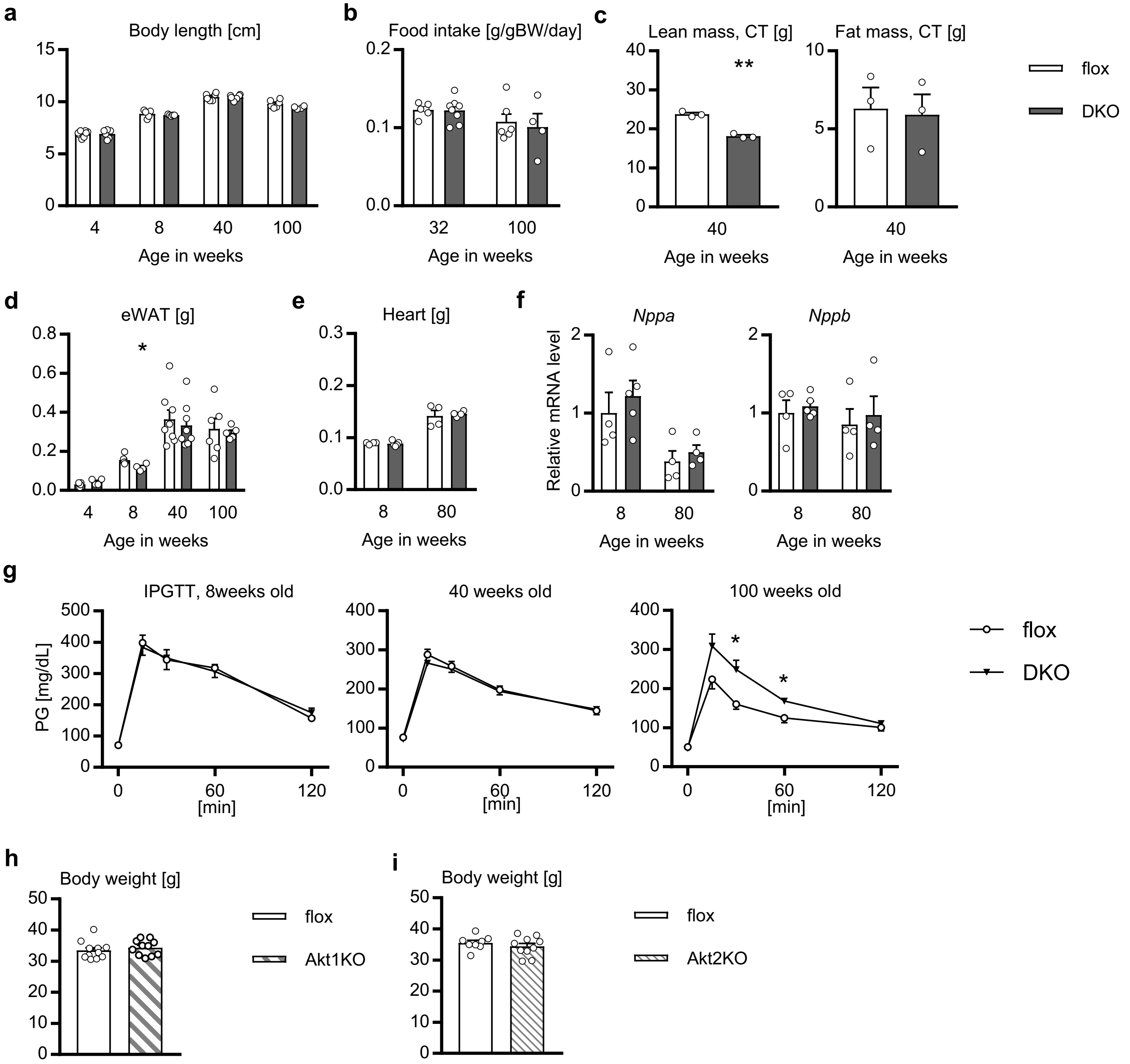

Supplementary Fig. 3 Phenotypes of the mAktDKO mice.

a,b, (**a**) Body length (n = 4, 5, 6, or 8 mice per group, as shown by the number of data points on the graph, same as below), and (**b**) food intake (n = 4, 6, or 8 mice), of the mAktDKO mice at the age of the indicated weeks.

c, Lean body mass and fat mass analyzed by CT scanning of the mAktDKO mice at the age of 40 weeks (n = 3 mice).

d, Tissue weight of the mAktDKO mice at the age of the indicated weeks (n = 4, 5, 6 or 8 mice). eWAT: epididymal white adipose.

e-f, (**e**) Heart weight, and (**f**) gene expression of natriuretic peptides in heart analyzed by RT-PCR, of the mAktDKO mice at the age of the indicated weeks (n = 4 or 5 mice).

g, Plasma glucose (PG) in glucose tolerance test (GTT) of the mAktDKO mice, after intraperitoneal injection of glucose (1.5 g/kg BW, 1.0 g/kg BW, and 2.0 g/kg BW, at the age of 8, 40, and 100 weeks, respectively) (n = 10 mice at the age of 8 weeks, n = 5 or 9 mice at the age of 40 weeks, and n = 4 or 9 mice at the age of 100 weeks).

h,i, (**h**) Body weight of, (**h**) the skeletal muscle-specific *Akt1* knockout mice at the age of 32 weeks (n = 11 mice), and (**i**) the skeletal muscle-specific *Akt2* knockout mice at the age of 32 weeks (n = 8 or 10 mice).

Values of the data are expressed as mean ± SEM. *P < 0.05, **P < 0.01. Unpaired 2-tailed t-test was used for assessment, and the exact P values are provided in Supplementary Data 3. Source data are provided as a Source Data file.

Supplementary Figure 4

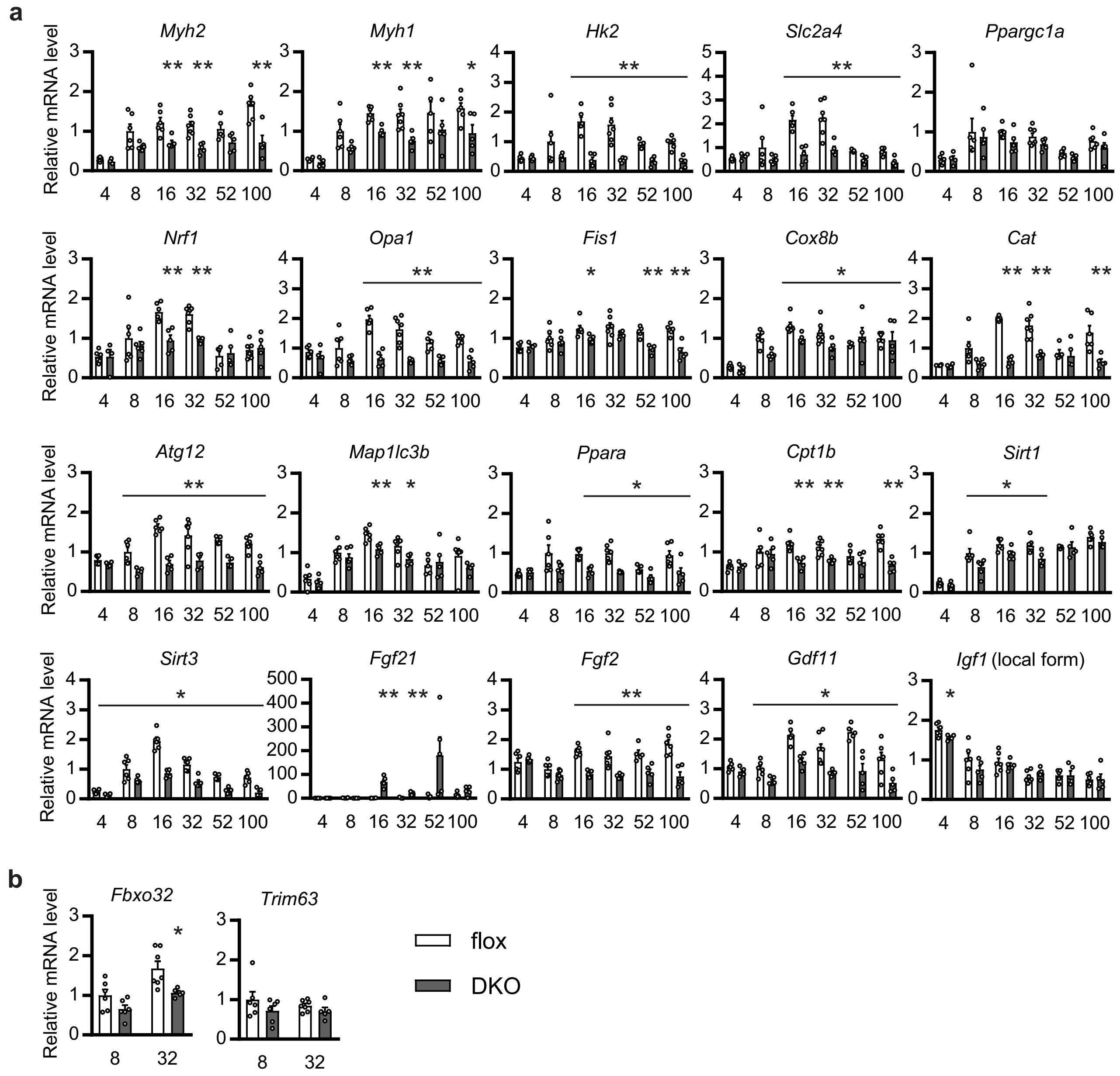

Supplementary Fig. 4 Changes in gene expression of fast-twitch muscle of the mAktDKO mice.

a,b, Gene expression of EDL of the mAktDKO mice at the age of the indicated weeks analyzed by RT-PCR ((**a**) n = 4, 5, 6 or 7 mice per group, and (**b**) n = 5, 6 or 7 mice, as shown by the number of data points on the graph).

Values of the data are expressed as mean ± SEM. *P < 0.05, **P < 0.01. Unpaired 2-tailed t-test was used for assessment, and the exact P values are provided in Supplementary Data 3. Source data are provided as a Source Data file.

Supplementary Figure 5

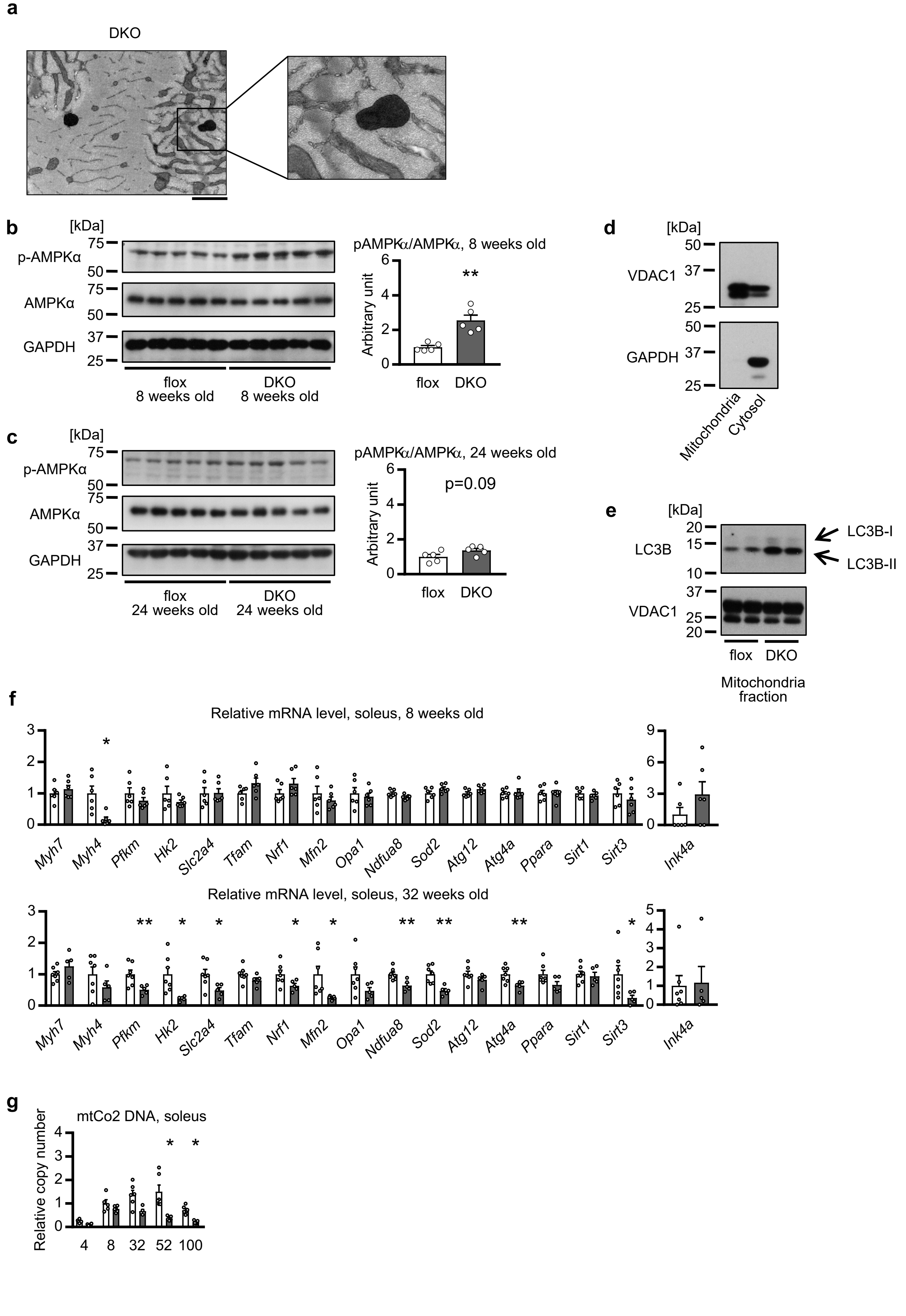

Supplementary Fig. 5 Mechanisms underlying the phenotypes of the mAktDKO mice.

a, Electron microscopic images of vertical sectioning of EDL of the mAktDKO mice at the age of 60 weeks, which was replicated in 3 mice. Scale bar: 1 μm.

b,c, Western blotting of gastrocnemius after exercise for, (**b**) 60 minutes at the age of 8 weeks, and (**c**) 15 minutes at the age of 24 weeks (n = 5 mice per group, as shown by the number of data points on the graph, same as below).

d,e, Representative images of western blotting of gastrocnemius, (**d**) to validate isolation of mitochondrial fraction in wild-type mice, and (**e**) to examine an autophagy-related protein in mitochondrial fraction at the age of 60 weeks. The experiments were repeated independently twice.

f,g, (**f**) Gene expression, and (**g**) DNA copy number, of soleus at the age of the indicated weeks analyzed by RT-PCR ((**f**) n = 6 mice at the age of 8 weeks, and n = 5 or 7 mice at the age of 32 weeks, and (**g**) n = 4, 5, or 6 mice).

Values of the data are expressed as mean ± SEM. *$P < 0.05$, **$P < 0.01$. Unpaired 2-tailed t-test was used for assessment, and the exact P values are provided in Supplementary Data 3. Source data are provided as a Source Data file.

Supplementary Figure 6

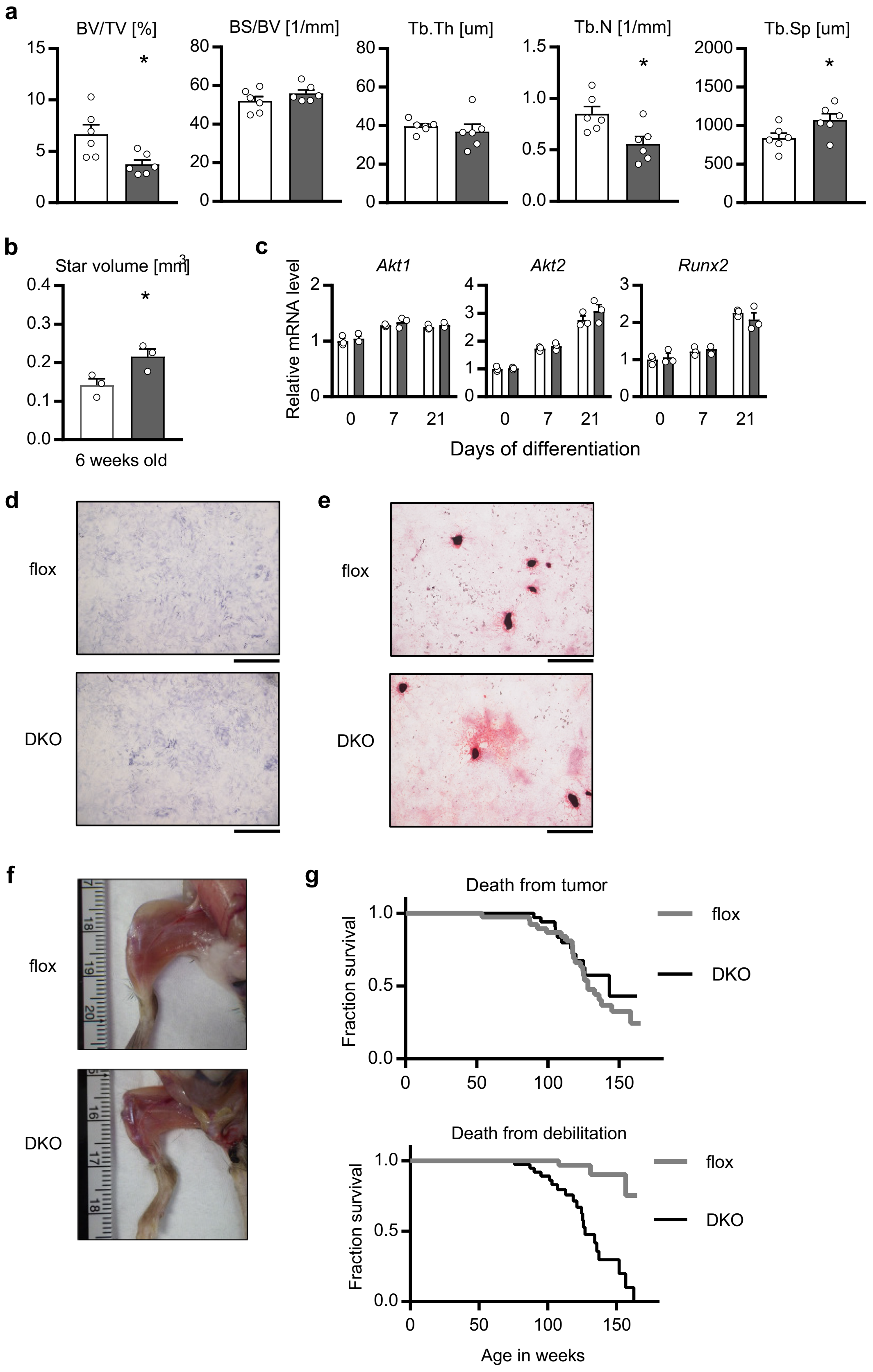

Supplementary Fig. 6 Bone-associated and systemic phenotypes of the mAktDKO mice.

a, Trabecular structure parameters analyzed by micro-CT scanning of femur of the mAktDKO mice at the age of 52 weeks (n = 6 mice per group, as shown by the number of data points on the graph, same as below). BV/TV: bone volume/tissue volume, BS/BV: bone surface/bone volume, Tb.Th: trabecular thickness, Tb.N: trabecular number, Tb.Sp: trabecular separation.

b, Analysis of osteoporosis by micro-CT scanning of femur at the age of 6 weeks (n = 3 mice).

c, Gene expression in isolated calvarial cells at the indicated days of differentiation to osteoblasts (n = 3 wells).

d,e, Analysis of isolated calvarial cells during differentiation by, (**d**) ALP staining at day 7, and (**e**) Alzarin Red staining at day 21. Scale bars: 1 mm.

f, Macroscopic image of lower limb of a dissected dead body.

g, Cause of death-specific Kaplan-Meire curves (n = 35 or 39 mice).

Values of the data are expressed as mean ± SEM. *P < 0.05. (**a-c**) Unpaired 2-tailed t-test was used for assessment, and the exact P values are provided in Supplementary Data 3. Source data are provided as a Source Data file.

Supplementary Figure 7

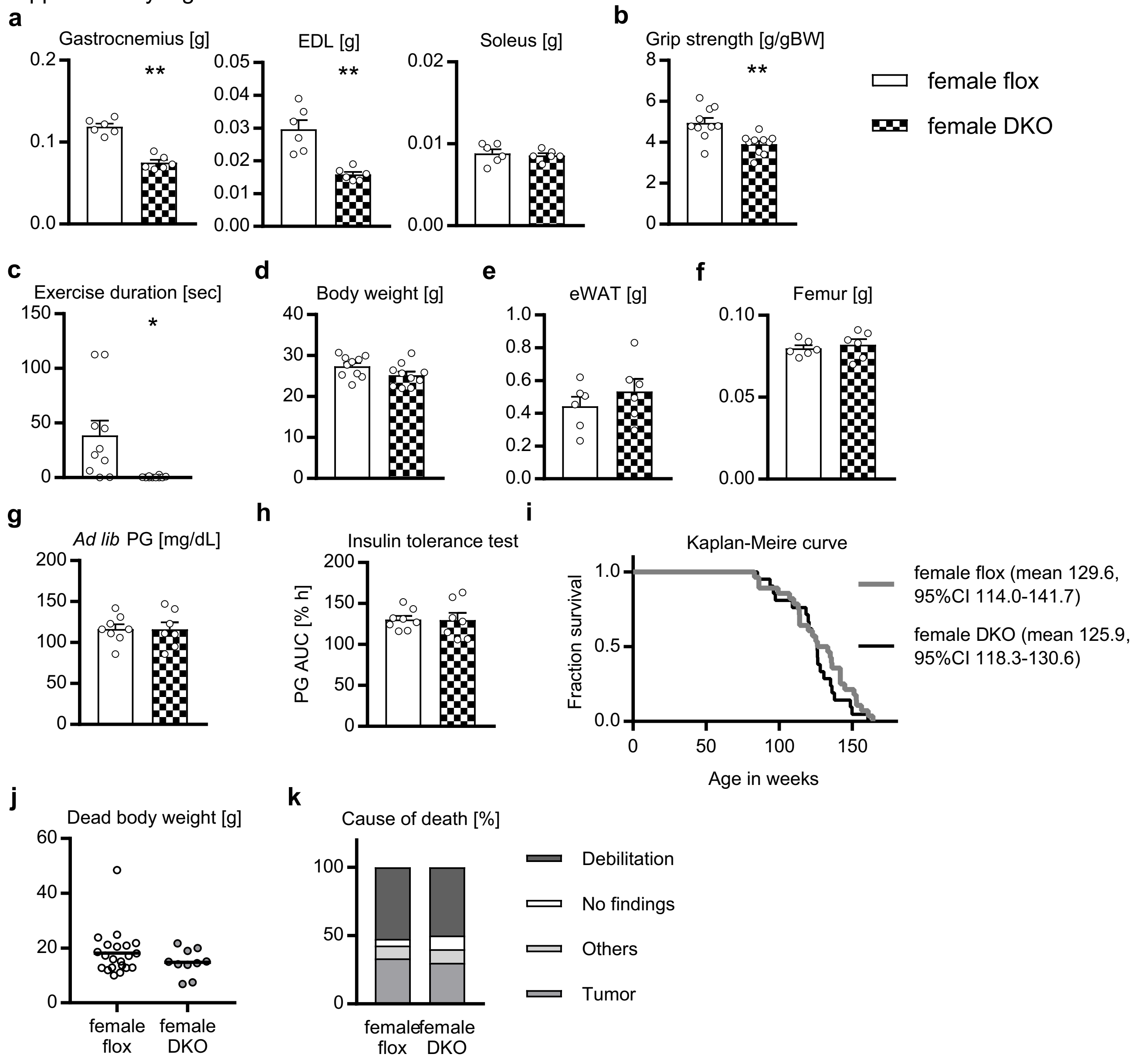

Supplementary Fig. 7 Phenotypes of the female mAktDKO mice.

a-f, (**a,e,f**) Tissue weight (n = 6 mice per group, as shown by the number of data points on the graph, same as below), (**b**) grip strength (n = 10 mice), (**c**) exercise duration (n = 10 mice), and (**d**) body weight (n = 10 mice), of the female mAktDKO mice at the age of 80 weeks. eWAT: epididymal white adipose.

g,h, (**g**) *Ad libitum* plasma glucose (PG) before insulin challenge, and (**h**) AUC of relative PG in ITT after intraperitoneal injection of human regular insulin (1.0 U/kg BW) at the age of 80 weeks (n = 7 or 8 mice).

i, Kaplan-Meire curve for survival (n = 21 or 28 mice).

j,k, (**j**) Scatter plot of dead body weight, and (**k**) cause of death, of mice whose dead body was retrieved without severe deterioration (n = 10 or 21 mice).

Values of the data are expressed as mean ± SEM. *P < 0.05, **P < 0.01. Unpaired 2-tailed t-test (**a-h,j**), logrank test (**i**), and Chi-square test with Yates' correction (**k**) were used for assessment, and the exact P values are provided in Supplementary Data 3. Source data are provided as a Source Data file.

Supplementary Figure 8

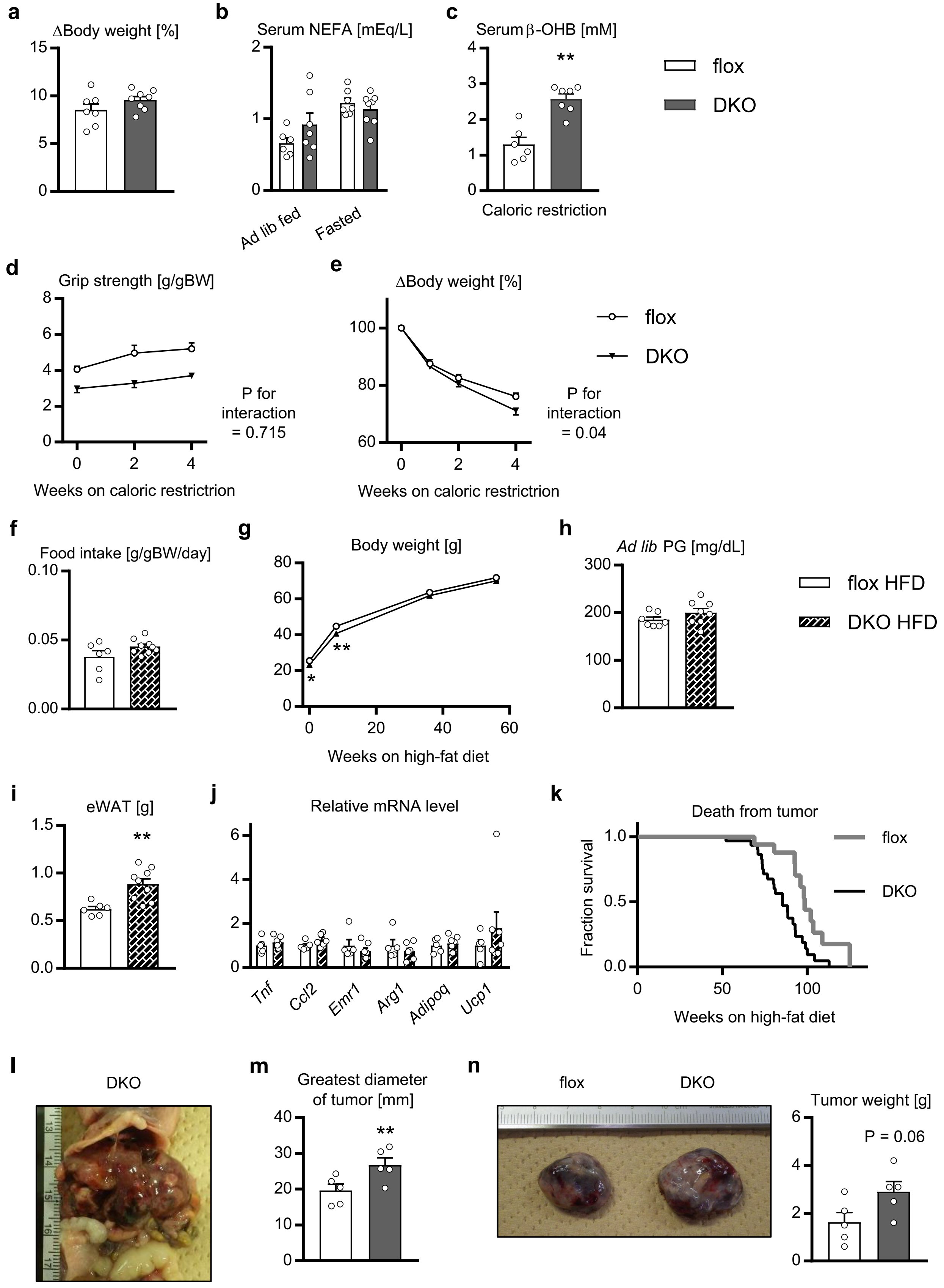

Supplementary Fig. 8 Phenotypes of the mAktDKO mice in under- and over-nutrition.

a,b, (**a**) Relative change in body weight during fasting for 24 hours (n = 7 or 8 per group, as shown by the number of data points on the graph, same as below), and (**b**) serum non-esterified fatty acid (NEFA) levels in the *ad libitum* fed state and in the fasted state for 24 hours (n = 6, 7 or 8 mice), of the mAktDKO mice at the age of 60 weeks.

c, Serum β-hydroxybutyrate (β-OHB) levels in the surviving mAktDKO mice at day 14 of caloric restriction (n = 6 or 7 mice).

d, Grip strength during caloric restriction of the surviving mAktDKO mice (n = 6 mice).

e, Relative change in body weight during caloric restriction of the surviving mAktDKO mice (n = 9 mice).

f, Food intake of the mAktDKO mice fed with high-fat diet for 40 weeks (n = 6 or 9 mice).

g, Body weight of the mAktDKO mice fed with high-fat diet, compared to the flox mice fed with high-fat diet, for the indicated weeks (n = 11 or 17 mice).

h, *Ad libitum* plasma glucose (PG) before insulin challenge of the mAktDKO mice fed with high-fat diet for 40 weeks (n = 7 or 8 mice).

i,j, (**i**) Weight (n = 6 or 9 mice), and (**j**) gene expression analyzed by RT-PCR (n = 5 or 7 mice), of epididymal white adipose tissue (eWAT) of the mAktDKO mice fed with high-fat diet for 40 weeks.

k, Cause of death-specific Kaplan-Meire curve for survival of the mAktDKO mice fed with high-fat diet (n = 16 or 28 mice).

l, Macroscopic image of liver tumor of a dissected mAktDKO mice.

m,n, (**m**) The greatest diameter of tumor, and (**n**) weight of excised tumor, were measured 3 weeks after subcutaneous transplantation of B16F1 cells (1x10^6 cells/body) into the mAktDKO mice at the age of 90 weeks (n = 5 mice).

Values of the data are expressed as mean ± SEM. *P < 0.05, **P < 0.01. HFD: high-fat diet. Unpaired 2-tailed t-test (**a-c,f-j,m,n**) and repeated measure analysis of variance (**d,e**) were used for assessment respectively, and the exact P values are provided in Supplementary Data 3. Source data are provided as a Source Data file.

Supplementary Figure 9

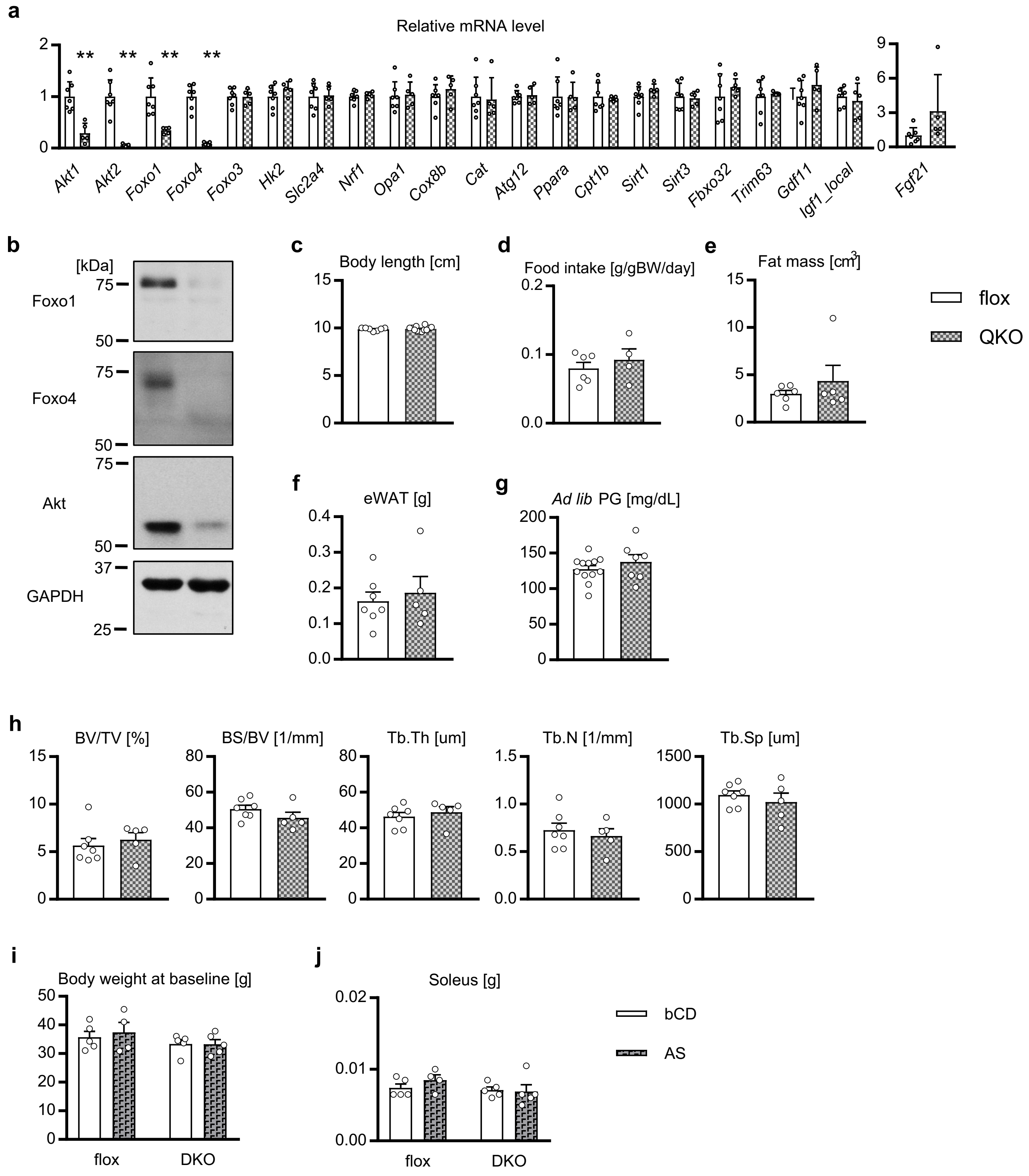

Supplementary Fig. 9 Phenotypes of the mAkt/FoxoQKO mice.

a, Gene expression of EDL of the mAkt/FoxoQKO mice at the age of 52 weeks analyzed by RT-PCR (n = 5 or 7 mice per group, as shown by the number of data points on the graph, same as below).

b, Representative images of western blotting to analyze expression of FoxO and Akt proteins in gastrocnemius of the mAkt/FoxoQKO mice at the age of 8 weeks. The experiments were repeated independently twice.

c, Body length of the mAkt/FoxoQKO mice at the age of 56 weeks (n = 7 or 12 mice).

d, Food intake of the mAkt/FoxoQKO mice at the age of 40 weeks (n = 4 or 6 mice).

e, Fat mass of the mAkt/FoxoQKO mice at the age of 52 weeks analyzed by systemic CT scanning (n = 5 or 6 mice).

f, Adipose tissue weight of the mAkt/FoxoQKO mice at the age of 52 weeks (n = 5 or 7 mice). eWAT: epididymal white adipose.

g, *Ad libitum* plasma glucose (PG) before insulin challenge of the mAkt/FoxoQKO mice at the age of 40 weeks (n = 7 or 12 mice).

h, Trabecular structure parameters analyzed by micro-CT scanning of femur of the mAkt/FoxoQKO mice at the age of 52 weeks (n = 5 or 7 mice). BV/TV: bone volume/tissue volume, BS/BV: bone surface/bone volume, Tb.Th: trabecular thickness, Tb.N: trabecular number, Tb.Sp: trabecular separation.

i,j, (**i**) Body weight at baseline, and (**j**) skeletal muscle weight, of the mAktDKO mice at the age of 90 weeks treated with AS1842856 at the dose of 100 mg/kg of BW for 4 weeks (n = 4 or 5 mice). bCD: β-cyclodextrin, AS: AS1842856.

Values of the data are expressed as mean ± SEM. $^{**}P < 0.01$. Unpaired 2-tailed t-test was used for assessment respectively, and the exact P values are provided in Supplementary Data 3. Source data are provided as a Source Data file.

Supplementary Figure 10

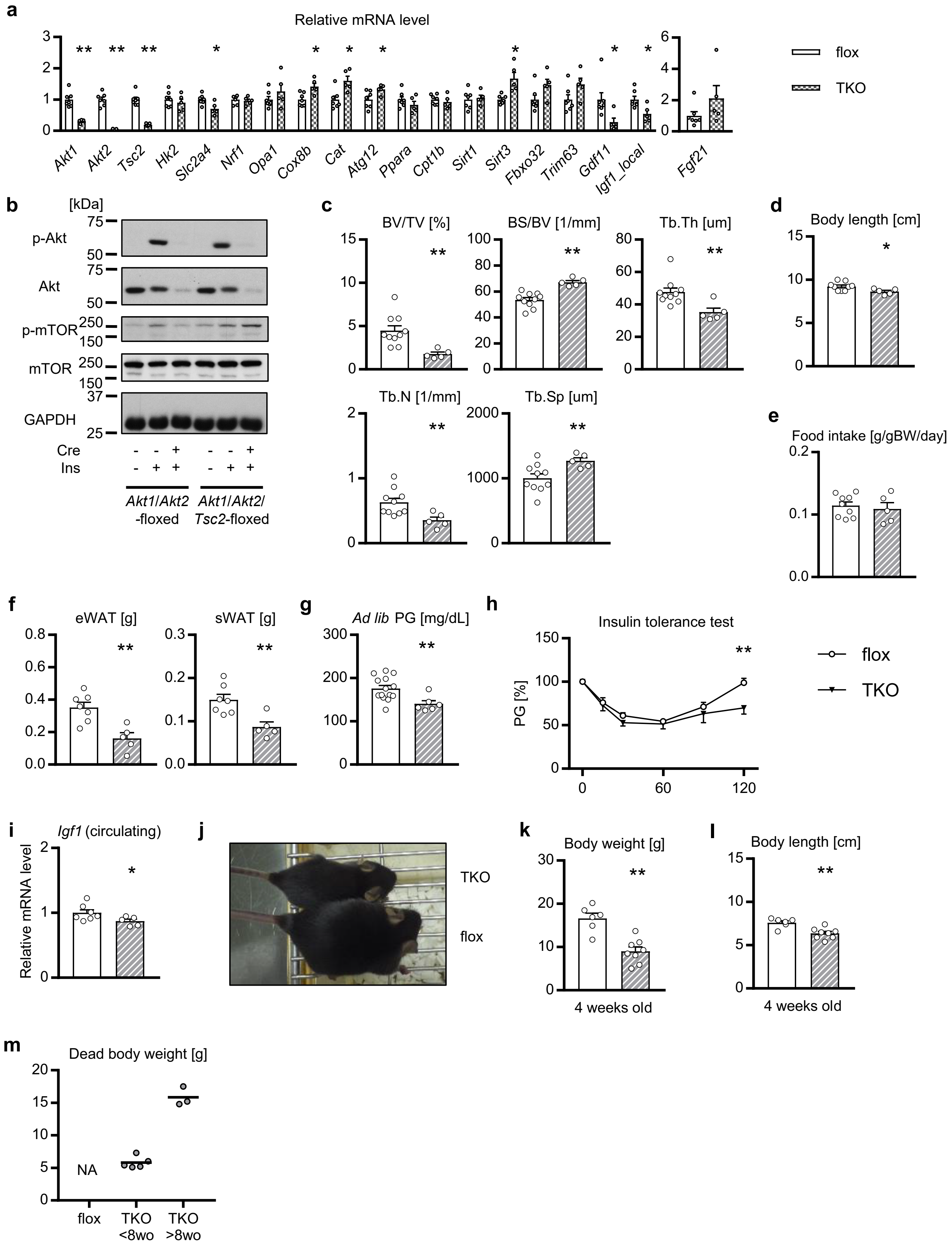

Supplementary Fig. 10 Phenotypes of the mAkt/TscTKO mice.

a, Gene expression of EDL of the mAkt/TscTKO mice at the age of 52 weeks analyzed by RT-PCR (n = 5 or 7 mice per group, as shown by the number of data points on the graph, same as below).

b, Representative images of western blotting to analyze insulin signaling in gastrocnemius at the age of 8 weeks. The experiments were repeated independently twice.

c, Trabecular structure parameters analyzed by micro-CT scanning of femur at the age of 52 weeks (n = 5 or 10 mice). BV/TV: bone volume/tissue volume, BS/BV: bone surface/bone volume, Tb.Th: trabecular thickness, Tb.N: trabecular number, Tb.Sp: trabecular separation.

d-f, (**d**) Body length (n = 5 or 9 mice), (**e**) food intake (n = 5 or 9 mice), and (**f**) tissue weight (n = 5 or 7 mice), at the age of 52 weeks. eWAT: epididymal white adipose tissue, sWAT: subcutaneous white adipose tissue.

g,h, (**g**) *Ad libitum* plasma glucose (PG) before insulin challenge, and (**h**) relative PG in ITT after intraperitoneal injection of human regular insulin (1.0 U/kg BW) at the age of 40 weeks (n = 6 or 14 mice).

i, Gene expression of the liver at the age of 52 weeks analyzed by RT-PCR (n = 5 or 7 mice).

j-l, (**j**) Macroscopic image of whole body, (**k**) body weight (n = 6 or 8 mice), (**l**) body length (n = 6 or 8 mice), at the age of 4 weeks.

m, Scatter plot of dead body weight of mice whose dead body was retrieved without severe deterioration (n = 3 or 5 mice). TKO <8wo: TKO mice dying earlier than 8 weeks, TKO >8wo: TKO mice surviving 8 weeks.

Values of the data are expressed as mean ± SEM. *P < 0.05, **P < 0.01. Unpaired

2-tailed t-test was used for assessment respectively, and the exact P values are provided in Supplementary Data 3. Source data are provided as a Source Data file.

Supplementary Figure 11

1a

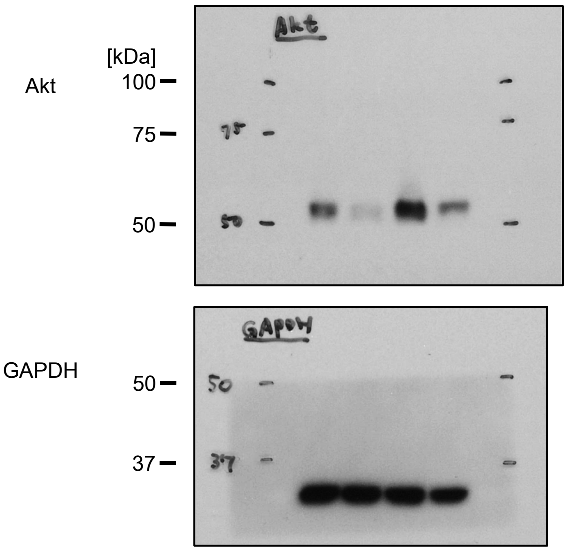

1c

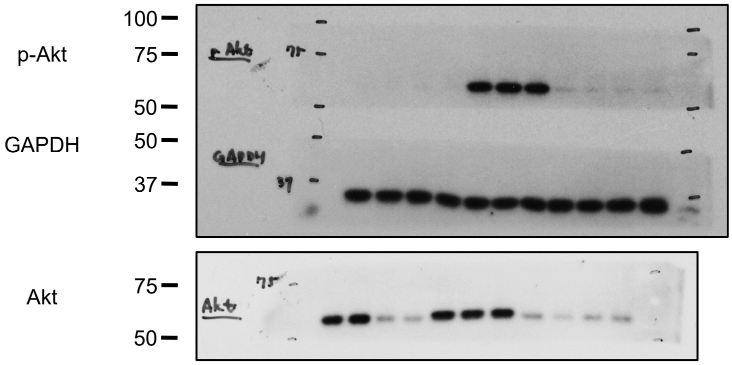

1d

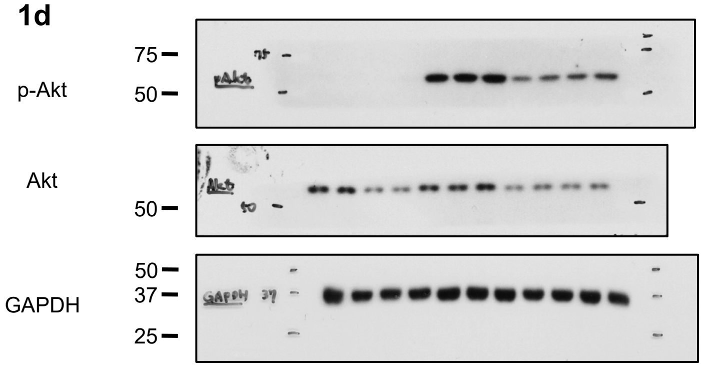

4f

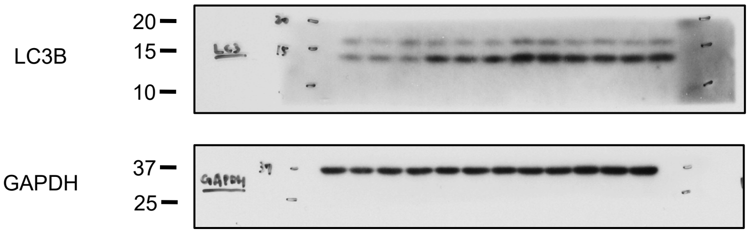

S1e

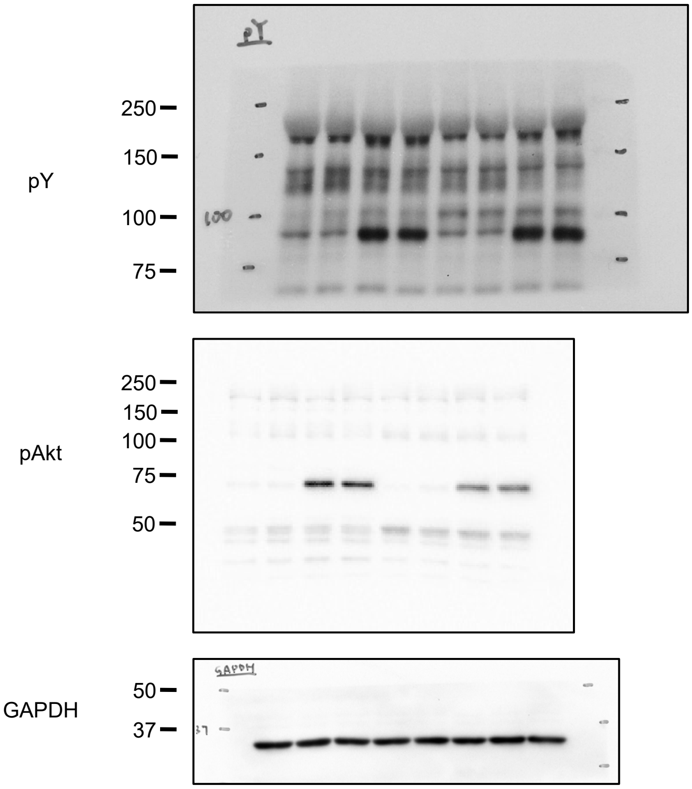

Supplementary Figure 11, continued

S2b

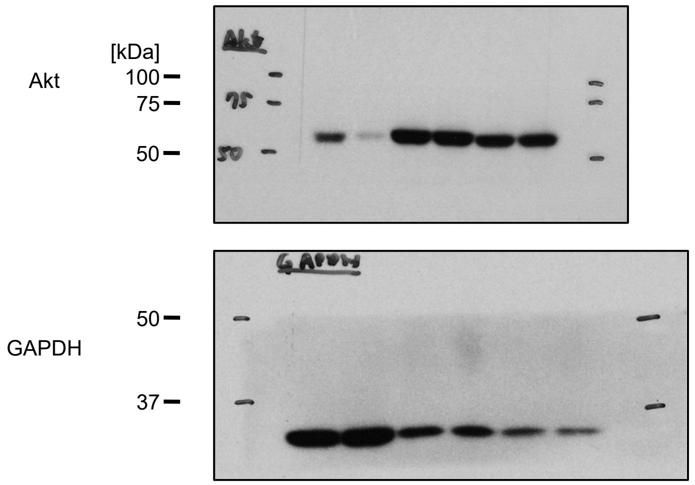

S2c

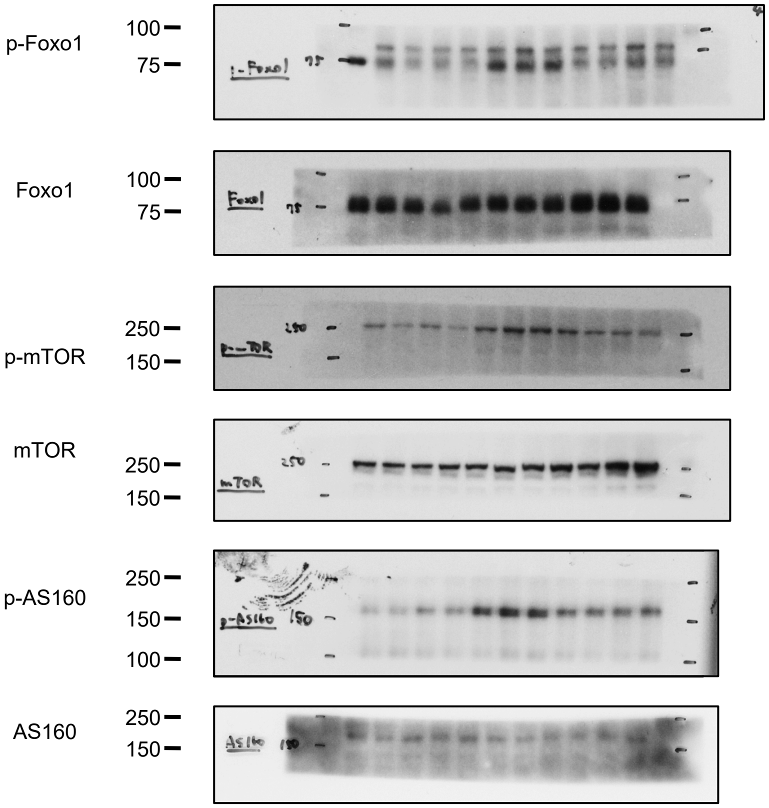

Supplementary Figure 11, continued

S2d

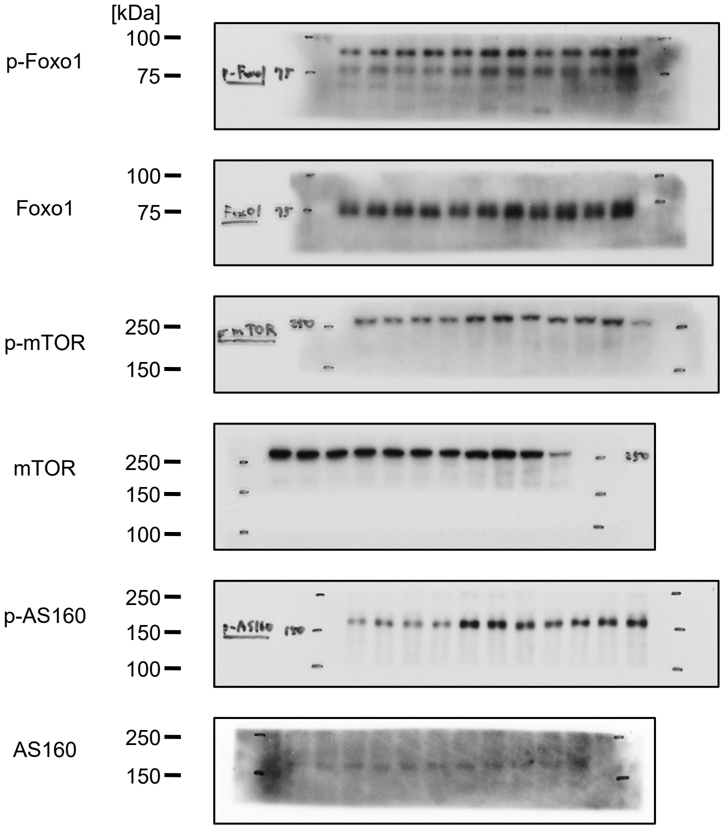

S5b

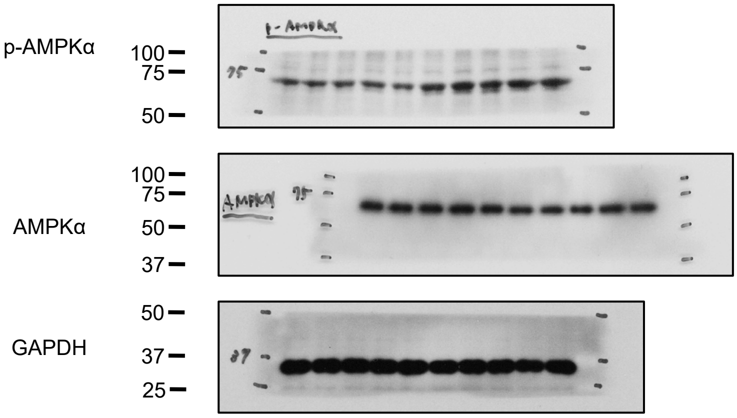

S5c

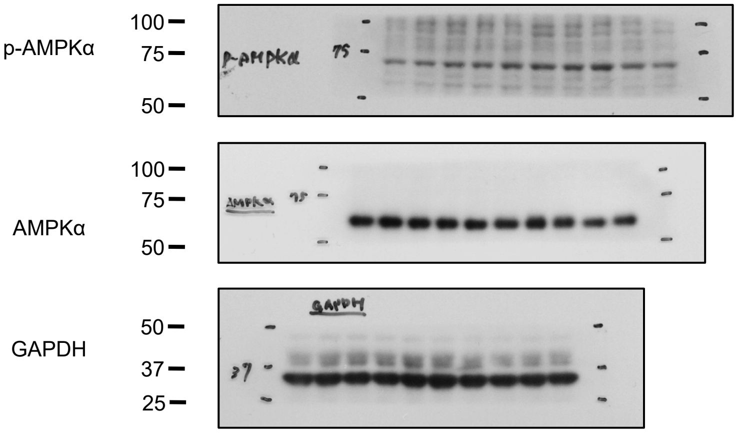

S5d

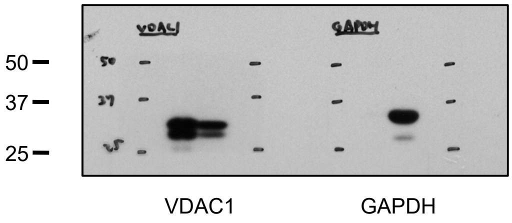

S5e

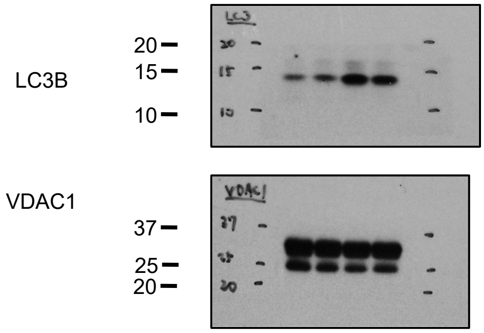

Supplementary Figure 11, continued

S9b

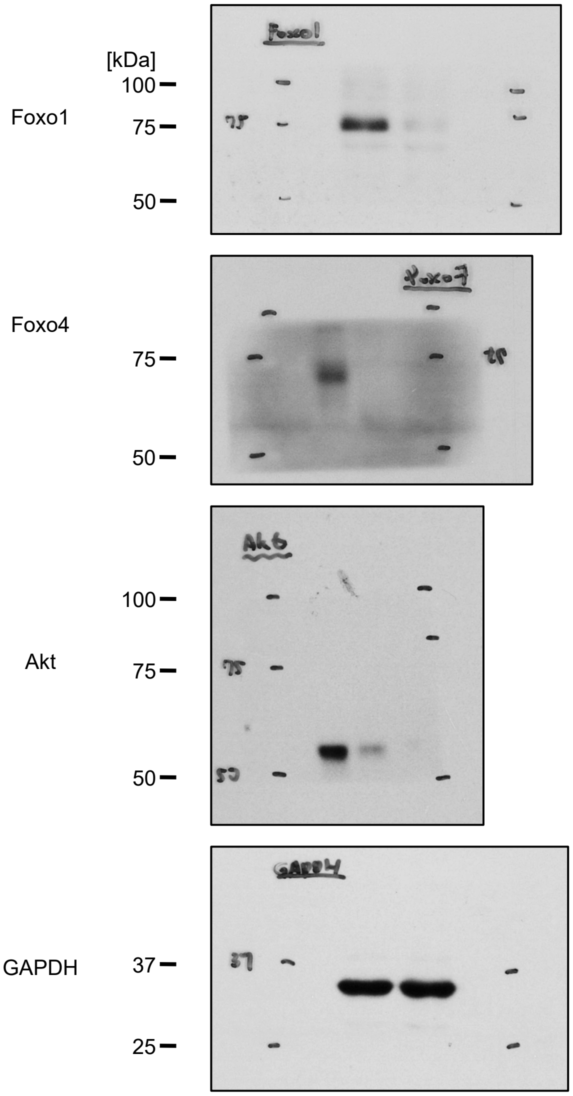

S10b

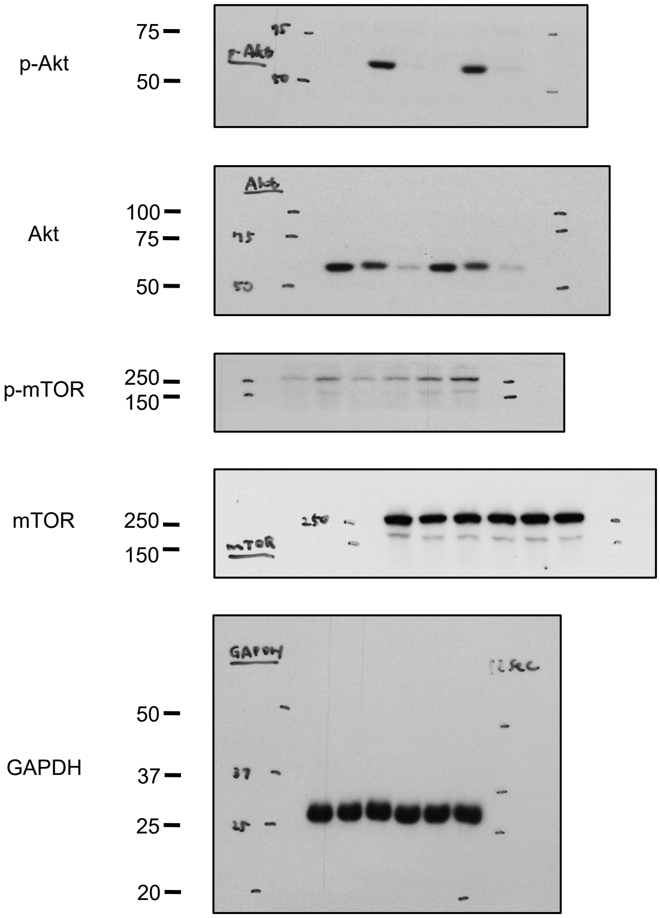

Supplementary Fig. 11 Uncropped and unprocessed scans of gels for western blotting.
