# Supplementary material for: Deletion of skeletal muscle Akt1/2 causes osteosarcopenia and reduces lifespan in mice
Source: Nat Commun. 2022 Oct 5;13:5655. doi: 10.1038/s41467-022-33008-2 (PMC9535008; doi:10.1038/s41467-022-33008-2)
Supplement: Supplementary file 3 — Description of Additional Supplementary Files [file 41467_2022_33008_MOESM3_ESM.pdf]

Description of Additional Supplementary Files

Supplementary Data 1: Results of transcriptome analysis of fast-twitch muscle of the mAktDKO mice.

Supplementary Data 2: P values of data shown in Figures.

Supplementary Data 3: P values of data shown in Supplementary Figures.
